# Role of non-native electrostatic interactions in the coupled folding and binding of PUMA with Mcl-1

**DOI:** 10.1371/journal.pcbi.1005468

**Published:** 2017-04-03

**Authors:** Wen-Ting Chu, Jane Clarke, Sarah L. Shammas, Jin Wang

**Affiliations:** 1 State Key Laboratory of Electroanalytical Chemistry, Changchun Institute of Applied Chemistry, Chinese Academy of Sciences, Changchun, Jilin, China; 2 Department of Chemistry, University of Cambridge, Lensfield Road, Cambridge, United Kingdom; 3 Department of Biochemistry, University of Oxford, South Parks Road, Oxford, United Kingdom; 4 Department of Chemistry & Physics, State University of New York at Stony Brook, Stony Brook, New York, United States of America; Max Planck Institute for Biophysical Chemistry, GERMANY

## Abstract

PUMA, which belongs to the BH3-only protein family, is an intrinsically disordered protein (IDP). It binds to its cellular partner Mcl-1 through its BH3 motif, which folds upon binding into an *α* helix. We have applied a structure-based coarse-grained model, with an explicit *Debye*—*Hückel* charge model, to probe the importance of electrostatic interactions both in the early and the later stages of this model coupled folding and binding process. This model was carefully calibrated with the experimental data on helical content and affinity, and shown to be consistent with previously published experimental data on binding rate changes with respect to ionic strength. We find that intramolecular electrostatic interactions influence the unbound states of PUMA only marginally. Our results further suggest that intermolecular electrostatic interactions, and in particular non-native electrostatic interactions, are involved in formation of the initial encounter complex. We are able to reveal the binding mechanism in more detail than is possible using experimental data alone however, and in particular we uncover the role of non-native electrostatic interactions. We highlight the potential importance of such electrostatic interactions for describing the binding reactions of IDPs. Such approaches could be used to provide predictions for the results of mutational studies.

## Author summary

Intrinsically disordered proteins (IDPs) are typically enriched in charged residues, with a general “coupled folding and binding” mechanism. We uncover the importance of non-native long-range electrostatic interactions between PUMA and Mcl-1 in the early stage of binding, by using the coarse-grained molecular dynamic simulations with charged model, which reveals the reason of the obviously increased binding rate at low ionic strength. However, intramolecular electrostatic interactions within PUMA can not influence the folding extent of unbound states of PUMA significantly. The results of binding-folding energy landscapes support an “induced fit” mechanism of binding. The critical residues and interactions between PUMA and Mcl-1 suggested here will be helpful for further mutational and mechanism studies.

## Introduction

The discovery of intrinsically disordered proteins (IDPs) [1–3], requires a reconsideration of the principles of protein-protein interactions, which have largely been examined using well-defined structured proteins. IDPs are involved in many critical physiological processes, in particular within protein interaction networks such as transcriptional and translational regulation, cellular signal transduction, protein phosphorylation, and molecular assembly [4]. Whilst disordered/unstructured at physiological conditions, IDPs that play such roles often undergo conformational changes to upon binding to their biomacromolecular partners [4, 5] in a process known as “coupled folding and binding” [5].

In the cell correct folding and binding of many proteins is essential for biological function. Since the 1890s the dominant descriptions of binding mechanisms are based on those described by Fischer: “lock and key” [6], “induced fit” [7] and “conformational selection” [8] (flexible binding). The lock and key mechanism, which involves rigid binding, is excluded for reactions that couple folding and binding. Recent kinetic studies have started to investigate the applicability of the “induced fit” and “conformational selection” mechanisms, which differ in whether the ligand is folded before or after binding.

Electrostatic interactions are known to play important roles in binding processes [9–11]. However so far only a limited number of experiments have investigated these interactions within coupled folding and binding reactions [12–14]. One of the most extensively experimentally studied coupled folding and binding processes is that between PUMA and Mcl-1. It is already known that electrostatic interactions are involved in association of PUMA with Mcl-1 as association rates are affected by salt concentrations [12]. Here we study the process of coupled folding and binding of PUMA with Mcl-1 using coarse-grained simulations that are specifically designed to include contributions from electrostatic interactions to uncover the nature of their involvement.

Mcl-1 is a stable folded pro-survival Bcl-2 protein that plays a critical role in development and tissue homeostasis. It is expressed in a range of tissue types, and is induced by a variety of stimuli to block apoptosis [15]. Gene knockout studies have demonstrated that Mcl-1 is required for embryonic and immune cell development [16, 17], while its over-expression is implicated in cancer and resistance to cancer treatments [15]. Pro-apoptotic BH3-only proteins such as PUMA, many of which are intrinsically disordered in their unbound state [18], form *α*-helices upon binding to Mcl-1.

Molecular dynamic simulations can gain many essential characteristics of IDPs that can not be obtained in experiments. However, the results of IDPs simulations are strongly influenced by the force field used [19–22]. Structure-based modeling can be a powerful tool for understanding coupled binding and folding of IDPs [23–27]. Classical structure-based models (SBMs) are established based on the native protein structure according to the conceptual framework of minimally frustrated energy landscapes. Therefore, the accuracy of IDP simulations is strongly dependent on parameters of SBM in the force field [27, 28]. Simple SBMs, with only a single energy basin, may not be appropriate for the systems of IDP complexes or systems with many conformational states of similar energy. As a result, we applied an advanced SBM based on the stable IDP complex structure, and treated IDP and folded protein differently. For the accuracy of the simulations, we carefully recalibrated the strengths of intramolecular and intermolecular interactions based on the experimental structural characteristics of IDP in solution and the binding affinity of the IDP complex, which have been successfully applied in many IDP complex systems [25, 27, 29, 30]. Besides, multi-basin models are also perfect choices to study the binding induced conformational changes of IDPs [31–34]. Here in this study, with a structure-based coarse-grained model [35–37] of the Mcl-1 and PUMA complex and a *Debye*—*Hückel* model to describe the electrostatic interactions at a moderate range of ionic strength, we investigate the contribution of ionic strength to the compactness of PUMA, and how this affects its rate of association with Mcl-1. Our results provide a residue level of detail for the mechanism of this modeled coupled folding and binding process, including the role of non-native electrostatic contacts in the formation of the encounter complex.

## Results

### Coupled folding and binding of PUMA with Mcl-1

**Fig 1:**
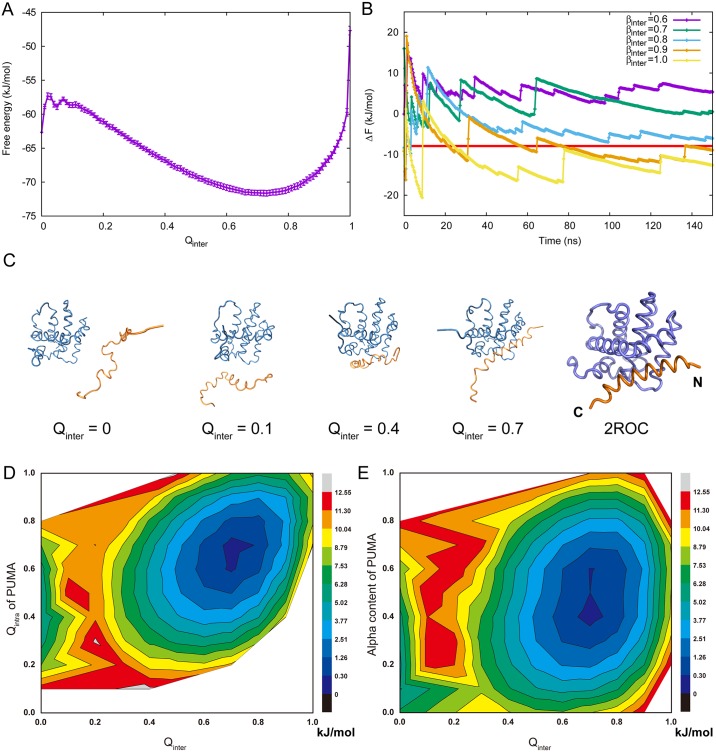
Parameter calibration and thermodynamic results. (A) Free energy profiles of Mcl-1 and PUMA complex using fraction of intermolecular native contact between Mcl-1 and PUMA (*Q*_*inter*_) as well as the standard deviation. The standard deviation is calculated with the free energy data of the last 10 ns. (B) The convergence of binding free energy of Mcl-1 and PUMA with different *β*. The binding free energy was calculated by the energy difference between bound state (*Q*_*inter*_ ∼ 0.7) and unbound state (*Q*_*inter*_ = 0). (C) Representative frames throughout the binding process. The bound state in our simulations is consistent with the NMR structure 2ROC (right). Mcl-1 and PUMA is depicted with dark blue and orange tubes, respectively. (D) Free energy landscape of Mcl-1·PUMA complex on both fraction of intermolecular native contact (*Q*_*inter*_) and fraction of intramolecular native contact of PUMA (*Q*_*intra*_). (E) Free energy landscape of Mcl-1 and PUMA complex on both fraction of intermolecular native contact (*Q*_*inter*_) and helix content of PUMA. The helix content is calculated using torison angles. The z axis is the free energy (kJ/mol) calculated by the probability using WHAM [40] without the effect of bias potential.

We aimed to describe the process of PUMA folding upon binding to its partner Mcl-1 in residue level detail using a simulation strategy that included consideration of electrostatic forces. To obtain the free energy landscape of the binding process between PUMA and Mcl-1, exchanges between unbound and bound states are needed in the thermodynamical simulations. Bias was added on the native contact pairs, aiming to enhance exchange rates between the unbound and bound states. After simulations, bias was removed to obtain the true free energy distribution. Firstly, the free energy of Mcl-1 and PUMA complex was projected on one reaction coordinate of intermolecular contact (*Q*_*inter*_) to provide an overview of the binding/unbinding process. A series of simulations were performed to calibrate the strength of intermolecular contacts (*β*). As shown in Fig 1B, when *β* = 0.9, the binding free energy of Mcl-1·PUMA complex is converged to about 7.93 *kJ*/*mol*, which matches the experimentally determined value of *K*_*d*_ [12, 38]. Using *β* = 0.9 we then investigated the nature of the coupled folding and binding process through two replicas of well-tempered metadynamics [39]. Fig 1A shows the free energy of the Mcl-1·PUMA complex as a function of the proportion of formed native intermolecular contacts (*Q*_*inter*_). There is a relatively small energy barrier of about 4 *kJ*/*mol* from the unbound state to the transition state, which is located at about 0.1 of *Q*_*inter*_. In contrast the energy of the system declines very sharply towards the bound state, which is located at about 0.72 *Q*_*inter*_. The bound state of Mcl-1·PUMA complex is so stable that the exchange between bound and unbound states is very slow (there are about only round 10 transitions from bound to unbound in each 128 ns simulation).

Snapshots of PUMA binding to Mcl-1 with various *Q*_*inter*_ are shown in Fig 1C to illustrate the process of binding although there are many potential structures for each value and simulations do not always involve the same contacts being formed first (see later).

The free energy landscape for coupled folding and binding was further examined using additional reaction coordinates to examine the nature of the process. Taken together intermolecular and intramolecular contact formation (*Q*_*inter*_ and *Q*_*intra*_) demonstrates a typical two-state system without populated intermediates (Fig 1) as has previously been suggested by experimental studies [12]. In its unbound state, the IDP PUMA is partly disordered with *Q*_*intra*_ about 0.4. Reflecting the coupling between folding and binding, within complex with Mcl-1 *Q*_*intra*_ of PUMA increases to about 0.7 (*Q*_*inter*_ about 0.72). Notably the *Q*_*intra*_ of PUMA does not change considerably between the unbound and transition states, demonstrating that the majority of PUMA folding takes places after binding i.e. via an induced fit mechanism. We previously postulated this mechanism after a *Φ*-value analysis for PUMA binding to Mcl-1 showed little structure formation was present in the transition state [30]. However as noted in the article experimental kinetic measurements are not able to determine mechanism unambiguously as we are able to here. With the aim of maximizing comparison of our results with those obtained previously through experiment we also considered an further reaction coordinate; the helical content of PUMA. In the bound state the IDP PUMA is folded as a part of regular alpha helix so *Q*_*intra*_ is related to the change of secondary structure of PUMA. Within the unbound and bound states the minima of the alpha helix content are about 10-20% and 40-60%, respectively. These results are consistent with the CD results of experiment [30]. In addition we are able to estimate the alpha helical content within the (unpopulated) transition state as approximately 18%.

### The effects of electrostatic interactions on unbound states of PUMA

**Fig 2:**
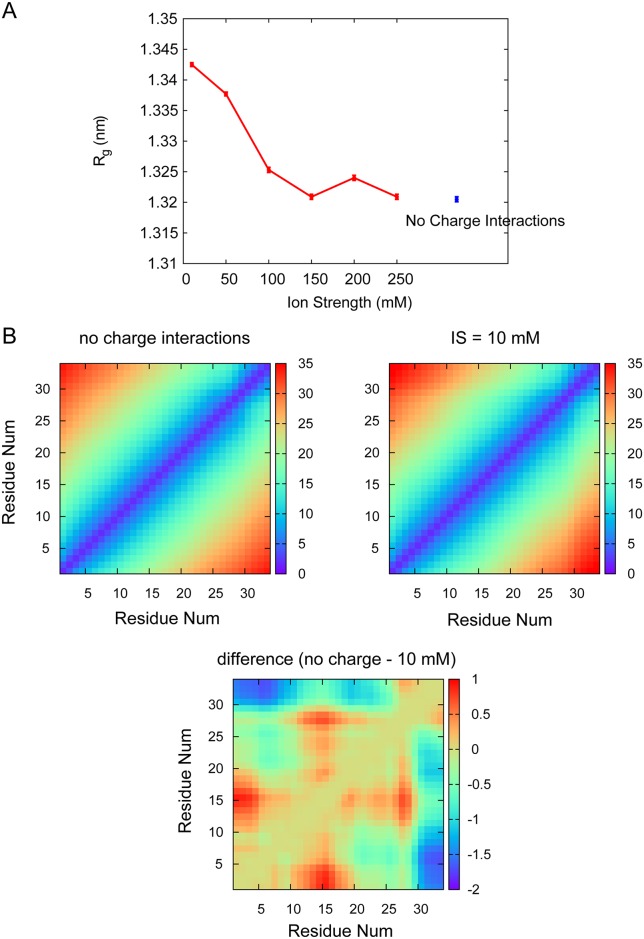
Radius of gyration and distance map results. (A) Average radius of gyration (*R*_*g*_) with standard error of unbound PUMA depends upon ionic strength. Simulations were performed using models of various ionic strengths from 10 mM to 250 mM (red dots). Blue dot indicates *R*_*g*_ obtained when charge-charge interactions are not included in the model. (B) Distance map of *C*_*α*_-*C*_*α*_ atoms of unbound PUMA with no charge-charge interactions (left) and high charge interactions of IS = 10 mM (right). The value of distance increases from 0 Å (blue) to 35 Å (red). The difference of residue-residue distance between the PUMA without charge interaction and PUMA in 10 mM salt solution is shown in (bottom).

IDPs are typically enriched in charged residues, and the PUMA BH3 domain is no exception. Within our 34 amino acid peptide there are 10 negatively charged residues and 5 positively charged residues (PUMA sequence is included in supplementary materials S1 Fig). Such an abundance of charged residues suggests that electrostatic interactions may play an important role in determining the structure of PUMA in its unbound ensemble. We therefore examined the effect of increasing the ionic strength upon the structure of unbound PUMA. High salt concentrations act to shield charges from each other and hence reduce intramolecular electrostatic interactions. We calculated the radius of gyration (*R*_*g*_) as a crude descriptor of the random coil ensemble of IDP PUMA. Fig 2 shows that *R*_*g*_ of PUMA does decrease with ionic strength, although the decrease is only small (from about 1.34 nm to 1.32 nm). Thus electrostatic interactions cause PUMA to be marginally more expanded than it would otherwise be. To probe this finding further we examined inter-residue distances within unbound PUMA. Distance maps (Fig 2B) reveal a lack of overall structural features. Instead inter-residue distance appears to be determined simply by separation distance in the amino acid sequence. This is highly consistent with its description as an IDP. However a more detailed examination demonstrates that electrostatic interactions modulate the inter-residue distances. Most notably, the distance maps for simulations with and without electrostatic interactions show that the distance between the two termini of PUMA increases slightly when charges are switched on. When electrostatic interactions are marginally shielded (IS = 10 mM) the distance between the two ends is about 2 Å larger when compared with no electrostatic interactions (Fig 2B). Other features are also apparent in the difference distance map, particularly for the arginine-containing region 12-18, which moves (up to) 1 Å closer to the negatively charged N-terminal region, and the region 18-30 when charges are introduced to the simulation.

**Fig 3:**
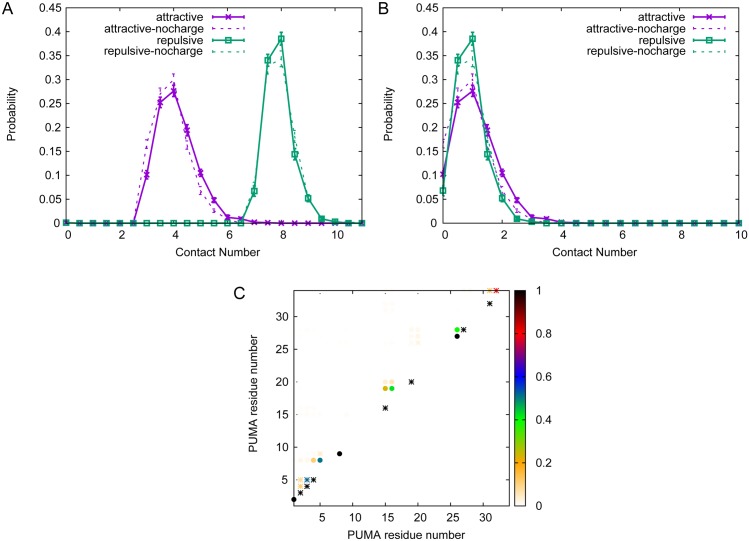
Contact information within PUMA. Repulsive charge contacts between directly connected residues dominate charge interactions within PUMA peptide. (A) the contact number distribution with standard deviation between all charged residues of PUMA i.e. between *i* and *j* atoms with ∣*i* − *j*∣≥ 1. (B) the contact number distribution with standard deviation between non-connected residues of PUMA i.e. between *i* and *j* atoms with ∣*i* − *j*∣> 1. Data of PUMA system in 10 mM ionic strength are shown with solid lines; data of PUMA system without charge interactions are shown with dashed lines. (C) the probability of each charge-charge interaction within PUMA in 10 mM ionic strength. Attractive and repulsive contacts are depicted by dots and asterisks, respectively.

We further probed the simulations for evidence of more local changes by examining residue-specific electrostatic interactions within unbound PUMA. The distance between head and tail of PUMA is very large, suggesting a long extended coil with no hairpins/turns (as shown in Fig 2B). The change of structure is largely related to the local interactions. We considered contacts made between oppositely and similarly charged residues i.e. both attractive and repulsive intra-peptide interactions. For structures, we considered intra-PUMA contacts as fully formed (contact number 1) if the distance between the “atoms” was under 4.5 Å. We considered intra-contacts as partially formed (contact number 0.5) if the distance was larger than 4.5 Å but under 6.0 Å. The distribution of contact numbers for the two interaction types shows that considerably more repulsive contacts (8 on average) are formed than attractive contacts (4 on average) (Fig 3A). However most of the contacts are between directly connected charged residues (black, Fig 3C) that must always be within 6.0 Å of each other. Excluding these there is an average of only one attractive and one repulsive contact being formed at any point (Fig 3B). Without electrostatic interactions, the probability of former opposite-charged contacts decreases, but the probability of former same-charged contacts increases. By contrast, the probability of intra-PUMA contacts when charges are switched off is shown in Fig 3A and 3B.

A contact map showing the probability of each individual interaction being present demonstrates that most of the charge contacts are between residues relatively close together within the PUMA sequence (Fig 3C and S2 Fig).

### The effects of electrostatic interactions on kinetics

**Fig 4:**
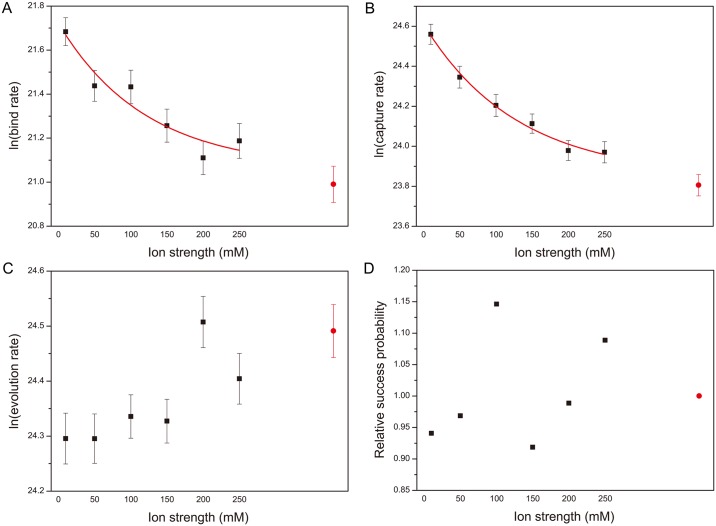
Role of electrostatic interactions in the binding process. Binding rate (A) and capture rate (B) for PUMA interacting with Mcl-1 at different ionic strengths depend upon ionic strength, whereas complex evolution rate (C) and relative success probability that proceed to bound complex (D) display little or no dependence upon ionic strength. Bind rate, capture rate, and evolution rate are obtained by calculating the reciprocals of *FPT*_*on*_, *MPT*_*cap*_ and *FPT*_*evo*_. All the units of rates are *s*^−1^. Red data points represent results from simulations performed with no charges included. Successful collision rate is calculated by mean value of each quotient of bind rate and capture rate. Relative success probability is the ratio of successful collision rate with successful collision observed without electrostatic interactions. Lines to guide the eye.

To reveal the role of electrostatic interactions on the binding behaviour of Mcl-1·PUMA complex, we performed kinetic simulations without explicit electrostatic interactions, and in the presence of electrostatic interactions at 6 different ionic strengths (from 10 to 250 mM). For each ionic strength 200 simulations with different initial velocities and structures were performed to ensure efficient sampling. The nature of our simulation approach meant that each simulation contained a single binding event. In the absence of electrostatic interactions this binding event took place on average 1330 ± 90 ps into each simulation, which is equivalent to a binding rate of (1.3 ± 0.1) × 10^9^ s^−1^. As shown in Fig 4 this rate is increased when electrostatic interactions are included, and in an ionic strength dependent manner. The mean time increases quickly between 10 and 50 mM ionic strength and plateaus at higher ionic strengths, approaching the time observed in the absence of electrostatic interactions. At 10 mM ionic strength the binding takes place around 2.0 times faster with a rate of (2.6 ± 0.1) × 10^9^ s^−1^.

To probe the basis of this phenomenon we then considered the binding process as two parts: an encounter (or capture) step and a further evolution step [41]. Broadly speaking binding rates could be modulated through changes in three separate key parameters: rate of formation of encounter complex (capture), rate of evolution from encounter complex to bound complex, and probability of progression from encounter complex to bound complex. We examined the dependence of each of these separate stages on the ionic strength.

PUMA formed multiple encounter complexes before successful binding occurred. The rate of capture events without electrostatic interactions is (2.2 ± 0.1) × 10^10^ s^−1^. Similarly to the binding rate, this is increased in the presence of electrostatic interactions in an ionic strength dependent fashion, with a 2.1-fold rate enhancement at 10 mM ionic strength, (4.6 ± 0.2) × 10^10^ s^−1^ (Fig 4). Thus the binding rate and capture rate have remarkably similar behaviour with ionic strength, suggesting that electrostatic forces enhance binding rates essentially entirely through altering collision rates. Consistent with this proportion of successful capture events appears independent of ionic strength (Fig 4D). The evolution rate is also relatively insensitive to electrostatic interactions, ranging only from (4.3 ± 0.2) × 10^10^ s^−1^ without electrostatic interactions to (3.6 ± 0.14) × 10^10^ s^−1^ at 10 mM ionic strength (Fig 4C).

**Fig 5:**
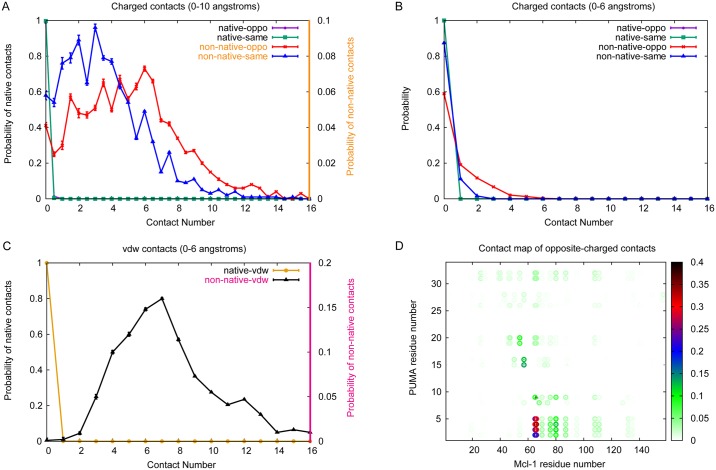
Contact number distribution and contact map at 10 mM ionic strength. Distribution of electrostatic and non-electrostatic interactions within the encounter complex (A-C). Contact frequencies for various interaction types within encounter complex (at 10 mM ionic strength). Standard errors of data are shown. (D) Observed opposite-charged contacts within encounter complex (at 10 mM ionic strength). Encounter complex structures were defined according to Huang *et al.* [41]. Native contacts are illustrated as triangles, non-native contacts are illustrated as circles.

**Fig 6:**
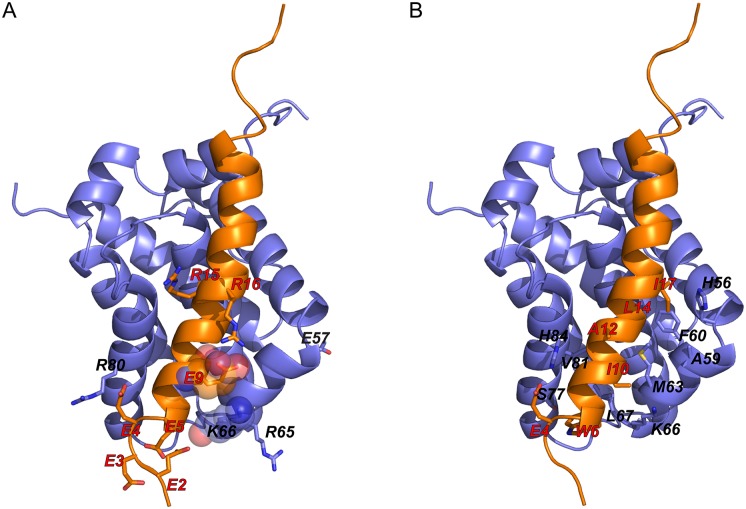
Critical residues within the encounter complex. Residues for opposite-charged (A) and vdw interactions (B) frequently observed within the encounter complex (probability over 0.1) at low ionic strength (10 mM). The one native opposite-charged contact pair with high probability is shown with spheres. Mcl-1 residues are labeled in black; PUMA residues are labeled in red.

For PUMA systems at low ionic strength (ionic strength = 10 mM), we have shown electrostatic interactions can accelerate the binding process. However there are very few native electrostatic intermolecular contacts within the encounter complex in our simulations (average number of contacts is only 0.1). However, the average of native charged contacts within 6 Å is 0 (Fig 5B), which suggests that the distances of the native charged contacts are all longer than 6.0 Å. The contact map of native opposite-charged contacts (Fig 5D) indicates that there is only one native opposite-charged contact with high probability, which is between residue Lys66 of Mcl-1 and Glu9 of PUMA (Fig 6A). In contrast there is a much wider distribution for the number of non-native opposite-charged contacts, with a much higher average contact number (5.1) than that for native contacts. This contact number is also somewhat higher than that for non-native same-charged contacts (3.5) (Fig 5A). The non-native charged interactions are almost all long-range (6.0—10.0 Å) (Fig 5A and 5B), and whilst it appears that many different interactions can be present in the encounter complex, some interactions are more likely than others. In particular interactions between the negatively charged residues in the N-terminal region of PUMA and Arg65/Lys66 of Mcl-1 are all present in over 20% of encounter complexes, and interactions between Arg15/Arg16 of PUMA and Glu57 of Mcl-1, and between Glu3/Glu4 of PUMA and Arg80 of Mcl-1 are present in over 10% of encounter complexes (see Figs 5D and 6A). Interestingly the N-terminal region of PUMA is also the region which we were previously able to show had formed some structure within the transition state for binding [30].

Therefore, PUMA firstly gets close to Mcl-1 with long-ranged electrostatic formed by its N-terminal residues (negatively charged) with positively charged residues (Arg65, Lys66, and Arg80) of Mcl-1.

There are more native non-electrostatic contacts (2.1) than native electrostatic contacts (0.1) in the encounter complex. Likewise, all the native non-electrostatic contacts are long-ranged (distance longer than 6.0 Å, Fig 5C). We note that there are also many non-native non-electrostatic interactions within the encounter complex (average 7.5, Fig 5C). However, none of the non-native non-electrostatic interactions has a probability > 0.05 (S3 Fig).

**Fig 7:**
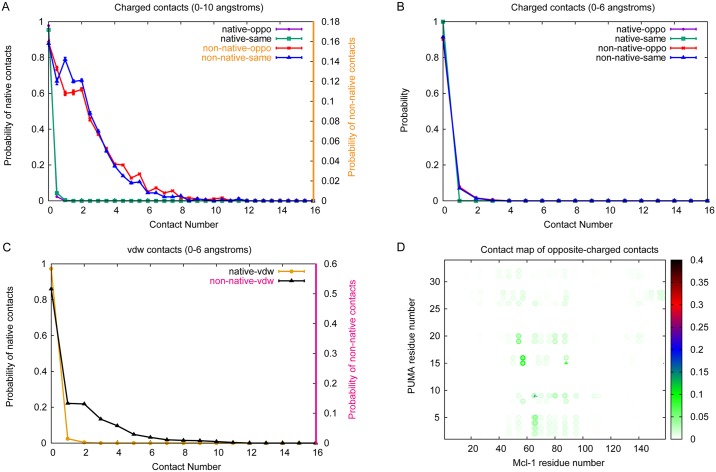
Contact number distribution and contact map without electrostatic interactions. Simulations in the absence of electrostatic forces indicate altered properties for the encounter complex. (A-C) Probability distributions of contact numbers within encounter complex. Standard errors of data are shown. (D) Contact map of opposite-charged contacts within encounter complex for simulations without electrostatic forces included. All other parameters are the same as simulations performed with charges (IS = 10 mM).

To dissect more fully the role of electrostatic forces from non-electrostatic ones in formation of the encounter complex we also performed simulations in the absence of charges for comparison. As previously very few native contacts were formed between previously charged residues. However the average number of non-native electrostatic contacts, both “opposite-charged” and “same-charged”, decreased to 2.1 and 1.9 respectively. The probability distribution of contact numbers has also changed so that the most likely number of contacts is 0 (Fig 7A and 7B), rather than 7 and 4 as obtained in simulations including electrostatics (10 mM). Most of the contacts formed are long-range. Examining the changes at the residue specific level indicates that this is mainly a result of a large decrease in “opposite-charged” contacts between Glu2/Glu3/Glu4/Glu5 of of PUMA and Mcl-1 (Fig 7D and S4A Fig), which will also affect the non-electrostatic interactions at this region (S4C Fig).

## Discussion

### Mechanism of PUMA folding upon Mcl-1 binding

We used coarse-grained molecular dynamics simulations to describe the mechanism of interactions between the natural intrinsically disordered PUMA BH3 peptide and its Bcl-2 partner protein Mcl-1. Our findings portray an induced fit binding process, with PUMA being relatively disordered at the transition state. This is in good agreement with previous experimental kinetic analyses that have suggested a relative unstructured transition state on the basis that the dissociation rate constant is much more sensitive to denaturant concentration than the association rate constant is [12]. Our simulations suggest the helical content of the transition state is approximately 18%, much closer to that of minima of the unbound state (10-20%) than the bound state (40-60%). This is highly consistent with, but extends, our knowledge of helical contents on the folding landscape from experimental studies that are fundamentally limited to populated states [30]. This compares favourably with Leffler *α* = 0.10 ± 0.01 determined from a recent Φ-value analysis, which aims to estimate the proportion of global structure formation at the transition state [30]. This work also showed the transition state to contain almost no structure at the C-terminus and only partial structure at the N-terminus [30]. This is highly consistent with the view formed of the encounter complex, formed before the transition state, from our simulations. These show the most probable contacts are formed between the N-terminus of PUMA with Mcl-1 residues that are close to its final position in the bound state.

### Role of electrostatic interactions in structure of unbound PUMA

Inter-residue distance maps of PUMA support its overall structural characterisation as an IDP since the largest determinant of the distance between any two residues is their sequence separation. Chebaro et al. [42] obtained a description of the energy landscape of PUMA and showed that the helix was unstable in the unbound state. The high proportion of charged residues within the PUMA sequence prompted us to examine whether structural changes might result from altering the ionic strength of the surrounding medium. Within our sequence of PUMA, there are 66 potential opposite-charged contact pairs and 70 potential same-charged contact pairs (including termini). The fraction of positive charged residues (*f*_+_) is 0.147 and the fraction of negative charged residues (*f*_−_) is 0.294. Then, the fraction of charged residues (FCR) and the net charge per residue (NCPR)/mean net charge are 0.441 and 0.147 (*FCR* = (*f*_+_ + *f*_−_); *NCPR* = |*f*_+_ − *f*_−_|), respectively. The mean hydrophobicity of PUMA is computed via the online tools ProtScale of ExPASy [43] (http://web.expasy.org/protscale/) with a window size of 5 residues and rescaled to fit between 0 and 1, which is 0.340. In addition, the intra-chain distance (*R*_*ij*_) has a relationship with chain separation (|*i* − *j*|), which can fit to the line of *y* = 0.331 *x*^0.683^. As shown in the Uversky diagram [44] and Das and Pappu diagram [45] of supplementary materials S5 Fig, PUMA is classified as the strong polyampholytes of IDPs [45–47]. Therefore, the characteristic of PUMA sequence is consistent with its behavior of *R*_*g*_ at different ionic strengths. However both measures of large-scale structural tendencies we employed, *R*_*g*_ and inter-residue distance maps, demonstrated only relatively small changes due to electrostatic interactions. Notably after 150 mM ionic strength there is no further decrease in radius, so the physiological situations may be well represented by simulations that do not include electrostatic contributions explicitly.

We are able to examine the subtle changes in PUMA structure in more detail. Unbounded PUMA is shown to be a extended coil. However the most significant contribution to the minor increase in PUMA radius when electrostatics are included in simulations, appears to be interactions between the two ends of the PUMA peptide. Both termini are negatively charged overall due to the presence of multiple glutamic acid residues (Glu2, Glu3, Glu4, Glu5, Glu31 and Glu32), and the difference inter-residue distance map indicates these move around 2 Å apart when charges are introduced and only slightly screened (IS = 10 mM). Indeed, the C-terminus (residue 30-34) moves further apart from the rest of the peptide in general.

We have shown that intra-chain electrostatic interactions within PUMA enlarge its radius. Thus at low ionic strengths the radius of interaction between Mcl-1 and PUMA may be increased. For induced fit processes such increased capture radii has been theoretically proposed to accelerate binding processes, a phenomenon termed the “fly-casting effect” [48, 49]. This phenomenon is thought to alter association rates less than 2-fold when comparing folded and random coil versions of the same protein. Thus small modifications in the radius such as those observed here may be expected to produce only tiny changes in association rates. Nonetheless we note that particular structural changes, especially those that alter the accessibility of charged residues, could impact on the efficiency of any “steering” of a protein towards its partner.

### Enhancement of association rates through electrostatics

Electrostatic interactions lead to both attractive and repulsive forces. Our simulations indicate that in the case of the PUMA⋅Mcl-1 complex the binding process is accelerated by electrostatic interactions i.e. attractive forces dominate. We observed that association rates decreased at higher ionic strengths where charges are more efficiently screened from each other and electrostatic interactions weakened. This behaviour is similar to that observed in many kinetic experiments of protein-protein association where association rates are found to vary depending upon the salt concentration. In fact such rate enhancement has already been observed experimentally for PUMA association with Mcl-1, though the effect was slightly more pronounced [12]. Whilst in experiment *k*_*on*_ decreases roughly 2-fold from 10 mM to 50 mM (about 9.8 × 10^7^ and 4.4 × 10^7^ *M*^−1^ *s*^−1^), in our simulations, the binding rate decreases only just over 1.3-fold over the same range. This may be due to the peptide differing by a single charged residue from that used in the experiments, or missing fine structural details as a result of our coarse-grained molecular dynamics approach.

Examining this behaviour through simulation allows us to separately consider the role of electrostatic interactions in capture and evolution parts. Our data suggest that electrostatic forces assist the initial formation of the encounter complex, and play a lesser role in subsequent progression to the bound state. Simulations from the group of Jianhan Chen for p27 with Cdk2/cyclin A [26], and for p53-TAD1 with TAZ2, HIF-1a with TAZ1, and NCBD with ACTR [50] all demonstrated that electrostatic forces act to reduce energy barriers between the various states, thus enhancing collision rates. In contrast to our findings they also suggested an important role of electrostatic contacts in progression from the collision complex to the fully bound state [26, 50]. In fact we observe a 1.2-fold decrease in evolution rate upon introducing partially screened charges to the simulation, suggesting electrostatic interactions are actually retarding progression from the encounter complex to the bound state. Nonetheless this change remains smaller than the effect upon binding and capture rates, and given that the evolution time remains appreciably lower than the timescale for binding this is unlikely to have a meaningful contribution to binding rates in our simulations.

### Non-native contacts dominate within the encounter complex

Since the effect of altering ionic strength is mainly on the long-range electrostatic interactions of capture process we investigated the nature of the charge interactions within the encounter complex. In our model of the Mcl-1·PUMA complex there are only 3 native opposite-charged contacts (between Lys66 of Mcl-1 and Glu9 of PUMA, Asp88 of Mcl-1 and Arg15 of PUMA, Arg95 of Mcl-1 and Asp19 of PUMA) and 3 native same-charged contacts (between Lys87 of Mcl-1 and Arg15 of PUMA, Asp88 of Mcl-1 and Asp19 of PUMA, Arg95 of Mcl-1 and Arg15 of PUMA). Rate constants for binding reactions between pairs of folded proteins have been described to depend strongly upon charges located in the binding interface [51]. As an intrinsically disordered protein that undergoes induced fit binding to Mcl-1, PUMA does not have a well-defined binding interface in its unbound state. Nonetheless if the native interfacial contacts are involved in electrostatic rate enhancement of the capture rate then they will be present within the encounter complex. In contrast we observed only one native opposite-charged interaction (between Lys66 of Mcl-1 and Lys9 of PUMA) with long-range distance (distance larger than 6 Å) in encounter complex, which indicates the entrance for PUMA. Most native interactions formed in encounter complex are non-electrostatic in nature.

Long-range electrostatic interactions do play an important role in the capture event though, as evidenced by the ionic strength dependence of the capture rate. Instead non-native opposite-charged interactions contribute a lot on the capture process. On average over 5 such contacts are formed in each encounter complex at 10 mM ionic strength. There is a wide distribution of probable contact numbers, ranging from 1 to 7, which reflects the non-uniformity of encounter complex structures. The most commonly observed opposite-charged contacts are between Arg65/Lys66/Arg80 of Mcl-1 and Glu2/Glu3/Glu4/Gl5 of PUMA. Each charged residue has been found to form multiple different intermolecular contacts within the encounter complex, however mapping these contacts onto the final structure demonstrates that contacts are more likely where residues are close to each other in the final structure. Similar coarse-grained simulations of the Mcl-1 and PUMA complex have been performed earlier by Rogers et al. [30]. The N-terminus was demonstrated to be important for the early stages of PUMA binding, even without electrostatic interactions. Here we show further that the non-native electrostatic interactions between N-terminus of PUMA and Mcl-1 can act as a “steering force” for binding, especially at low ionic strengths. A similar finding was reported recently for a peptide of RA-GEF2 binding to its PDZ domain partner where various non-native dynamic salt bridges were found within the encounter complex [52]. Further evidence for the importance of these contacts is that excluding electrostatic forces from our simulations alters the distribution of PUMA over the Mcl-1 surface in the encounter complex, with less focussed “charged interactions”.

## Materials and methods

### Initial model of simulation

NMR coordinates of Mcl-1 complexed with PUMA (PDB ID 2ROC [53]) were prepared for constructing the model. In this structure, the PUMA is 27 a.a. length. According to the experiments of Rogers *et al.* [12], a full-length PUMA (34 a.a.) was build by using Chimera software [54] (S1 Fig). The initial coarse-grained C_*α*_ structure-based model (SBM) of Mcl-1 and PUMA complex was generated using SMOG on-line toolkit, which included one bead on the C_*α*_ atom of each residue of the complex [35, 36, 55, 56]. This model contains Mcl-1 (162 residues) and PUMA (34 residues), 424 and 36 intramolecular contacts within each of them, as well as 77 intermolecular contacts between them. The native contact map was built by the Shadow Algorithm [55]. The potential energy function consists of both bonded and nonbonded terms. Additionally, we introduced the charge characterization into our SBM model to study the electrostatic interactions in this system. As a result, the potential energy form used in this study is given in the following equation:

$$\begin{array}{cc} V= & \sum_{bonds}\epsilon_{r}{\left(r-r_{0}\right)}^{2}+\sum_{angles}\epsilon_{\theta }{\left(\theta -\theta_{0}\right)}^{2}\\ & +\sum_{dihedral}K_{\phi }^{\left(n\right)}\left(1-\cos(n\times \left(\phi -\phi_{0}\right))\right)\\ & +\sum_{contacts}\epsilon_{ij}\left(5{\left(\frac{\sigma_{ij}}{r_{ij}}\right)}^{12}-6{\left(\frac{\sigma_{ij}}{r_{ij}}\right)}^{10}\right)\\ & +\sum_{non-contacts}\epsilon_{NC}{\left(\frac{\sigma_{NC}}{r_{ij}}\right)}^{12}+V_{Debye-H\ddot{u}ckel} \end{array}$$ (1)

In Eq 1, *ϵ*_*r*_ = 100 *ϵ*, *ϵ*_*θ*_ = 20 *ϵ*, $K_{\phi }^{\left(1\right)}=\epsilon$ and $K_{\phi }^{\left(3\right)}=0.5\epsilon$. The interaction strength of Lennard-Jones-type potential is proportional to the statistical potential reported for the residue types of *i* and *j* by Miyazawa and Jernigan to generate the flavored model. [57] Therefore, the coefficient of nonbonded contacts, *ϵ*_*ij*_ is set as follows:

$$\epsilon_{ij}=\left(\gamma \left(\frac{\epsilon_{ij}^{MJ}}{{\bar{\epsilon }}^{MJ}}-1\right)+1\right)$$ (2)

where $\epsilon_{ij}^{MJ}$ is the original MJ potential, ${\bar{\epsilon }}^{MJ}$ is the mean value of the entire set of MJ weights in the complex system, and *γ* is set to 1.0 corresponding to the “flavored model” [58]. As with similar strategies in previous studies [25, 27, 29], the native nonbonded potential can be separated into intramolecular and intermolecular terms, which should be rescaled according to the experimental data (see the part of Method in supplementary materials).

The electrostatic interaction is calculated by the *Debye*—*Hückel* model, which can quantify the strength of charge-charge attractions and repulsions at various salt concentrations:

$$V_{Debye-H\ddot{u}ckel}=\Gamma_{DH}\times K_{coulomb}B\left(\kappa \right)\sum_{i,j}\frac{q_{i}q_{j}\exp\left(-\kappa r_{ij}\right)}{\epsilon r_{ij}}$$ (3)

In Eq 3, *K*_*coulomb*_ = 4 *πϵ*_0_ = 138.94 *kJ*·*mol*^−1^·*nm*·*e*^−2^ is the electric conversion factor; *B*(*κ*) is the salt-dependent coefficient; *κ*^−1^ is the Debye screening length, which is directly influenced by the solvent ionic strength (IS)/salt concentration *C*_*salt*_ ($\kappa \approx 3.2\sqrt{C_{salt}}$); *ϵ* is dielectric constant, which is set to 80 during the simulations. Γ_*DH*_ is the energy scaled coefficient which aims to make the total energy balanceable. In our model, Lys and Arg have a positive point charge (+e), Asp and Glu have a negative point charge (-e). All the charges are placed on the *C*_*α*_ atoms. Besides the systems in altering ionic strengths, there is also a system with no electrostatic interactions. Under physiological ionic strengths (*C*_*salt*_ ∼ 0.15 *M*), *κ* is 1.24 *nm*^−1^, so we set Γ_*DH*_ = 0.535 in our simulations, so that *V*_*DH*_ for two opposite charged atoms located at a distance of 0.5 nm matches the native contact energy. More details of *Debye*—*Hückel* model can be found in these papers [34, 59–61].

### Analysis of contact formation

Our definition of native contact cutoff is the same as that used in many previous structure-based model simulations, and is based on the distances between the residues in the experimental structures. In contrast, there is no universal standard for the definition of the cutoffs of non-native contacts. It is important that we analyze the native and non-native interactions in a consistent manner. For intermolecular non-native contacts, we checked all the native distances of the native contacts. We find that in the native complex the average distances between two C*α* atoms in two chains that form a contact is about 8.6 Å. In addition, the minimum of native distance between two C*α* atoms in two chains is 4.9 Å. As a result, when analysing contact number within the encounter complex we considered a contact between Mcl-1 and PUMA as fully formed (contact number 1) if the distance between the “atoms” was less than or equal to 6.0 Å (about 1.2 times of lowest distance of native inter-chain contacts); we considered contacts as partially formed (contact number 0.5) if the distance was larger than 6.0 Å but under 10.0 Å (non-native contacts, about 1.2 times of mean distance of native inter-chain contacts) [38]. This method was chosen to allow the same way of counting contact number for both native and non-native interactions.

### Analysis of ionic strength dependence of rates

ln (bind rate), ln (capture rate), and ln (evolution rate) were obtained from *FPT*_*on*_, *MPT*_*cap*_, and *FPT*_*evo*_, respectively,

$$\ln k=\frac{1}{n}\sum_{i}^{n}\ln\left(\frac{1}{tau_{i}}\right)$$ (4)

here, *k* represents bind rate/capture rate/evolution rate, *tau* represents *FPT*_*on*_/*MPT*_*cap*_/*FPT*_*evo*_, and n is 200 runs at each ionic strength. Collision success probability is obtained from bind rate and capture rate,

$$collision\;success\;probability=\frac{{\left(\frac{bind\;rate}{capture\;rate}\right)}_{I}}{{\left(\frac{bind\;rate}{capture\;rate}\right)}_{0}}$$ (5)

where *I* is the ionic strength.

## Supporting information

S1 Fig Sequence and structure information of Mcl-1 and PUMA complexed model. Upper: the sequence of full-length PUMA (34 a.a.) used in the simulations. Positive charged and negative charged residues are colored in blue and red. The region of helix in bound state is shown in light green box. Lower: The Mcl-1 · PUMA complex with full-length PUMA, constructed based on the NMR structure 2ROC (27 a.a. PUMA). Mcl-1 and PUMA are shown in green and red cartoons, respectively.
(TIF)

S2 Fig The probability of each charge-charge interaction within PUMA without charge interactions. Attractive and repulsive contacts are depicted by dots and asterisks, respectively.
(TIF)

S3 Fig Contact map at 10 mM ionic strength. Contact map of opposite-charged (A), same-charged (B), and vdw (C) interactions within encounter complex at 10 mM ionic strength, as well as the contact map of opposite-charged interactions within the beginning of the evolution part (D). The beginning of evolution is collected for the complexes with only 1-2 number of inter contacts in the evolution part. The cutoff of the contact distance is 10.0 Å. Native contacts are illustrated as triangles, non-native contacts are illustrated as circles.
(TIF)

S4 Fig Contact map without electrostatic interactions. Contacts are more evenly distributed within the encounter complex for simulations performed without electrostatic forces included. Contact map of opposite-charged (A), same-charged (B), and vdw (C) interactions within encounter complex, as well as the contacp map of opposite-charged interactions within the beginning of the evolution part (D). All other parameters are the same as simulations performed with charges and IS = 10 mM.
(TIF)

S5 Fig Charged residue distribution and structure information of PUMA. Top left: Uversky diagram [44] of IDPs (under dashed line) and globular proteins (above dashed line), Top right: Das and Pappu diagram [45] of IDPs. Bottom: Intra-chain distance (*R*_*ij*_) profiles of PUMA with respect to chain separation. Theoretical polymer scaling limit and fitting function are labeled.
(TIF)

S1 File Additional information of methods. (PDF)

## References

[1] Wright PE, Dyson HJ. Intrinsically unstructured proteins: re-assessing the protein structure-function paradigm. J Mol Biol. 1999;293(2):321–331. 10.1006/jmbi.1999.3110 10550212

[2] Dunker AK, Lawson JD, Brown CJ, Williams RM, Romero P, Oh JS, et al Intrinsically disordered protein. J Mol Graph Model. 2001;19(1):26–59. 11381529 10.1016/s1093-3263(00)00138-8

[3] Uversky VN. Natively unfolded proteins: a point where biology waits for physics. Protein Sci. 2002;11(4):739–756. 10.1110/ps.4210102 11910019 PMC2373528

[4] Dyson HJ, Wright PE. Intrinsically unstructured proteins and their functions. Nat Rev Mol Cell Biol. 2005;6(3):197–208. 10.1038/nrm1589 15738986

[5] Dyson HJ, Wright PE. Coupling of folding and binding for unstructured proteins. Curr Opin Struct Biol. 2002;12(1):54–60. 10.1016/S0959-440X(02)00289-0 11839490

[6] Fischer E. Einfluss der Configuration auf die Wirkung der Enzyme. Eur J Inorg Chem. 1894;27(3):2985–2993. 10.1002/cber.18940270364

[7] Koshland D Jr. Application of a theory of enzyme specificity to protein synthesis. Proc Natl Acad Sci U S A. 1958;44(2):98–104. 10.1073/pnas.44.2.98 16590179 PMC335371

[8] Bosshard HR. Molecular recognition by induced fit: how fit is the concept? Physiology. 2001;16(4):171–173. 10.1152/physiologyonline.2001.16.4.171 11479367

[9] Fersht A. Structure and mechanism in protein science: a guide to enzyme catalysis and protein folding. Macmillan; 1999.

[10] Schreiber G, Fersht AR. Rapid, electrostatically assisted association of proteins. Nat Struct Mol Biol. 1996;3(5):427–431. 10.1038/nsb0596-427 8612072

[11] Vijayakumar M, Wong KY, Schreiber G, Fersht AR, Szabo A, Zhou HX. Electrostatic enhancement of diffusion-controlled protein-protein association: comparison of theory and experiment on barnase and barstar. J Mol Biol. 1998;278(5):1015–1024. 10.1006/jmbi.1998.1747 9600858

[12] Rogers JM, Steward A, Clarke J. Folding and binding of an intrinsically disordered protein: fast, but not “diffusion-limited”. J Am Chem Soc. 2013;135(4):1415–1422. 10.1021/ja309527h 23301700 PMC3776562

[13] Shammas SL, Travis AJ, Clarke J. Allostery within a transcription coactivator is predominantly mediated through dissociation rate constants. Proc Natl Acad Sci U S A. 2014;111(33):12055–12060. 10.1073/pnas.1405815111 25092343 PMC4143058

[14] Shammas SL, Travis AJ, Clarke J. Remarkably fast coupled folding and binding of the intrinsically disordered transactivation domain of cMyb to CBP KIX. J Phys Chem B. 2013;117(42):13346–13356. 10.1021/jp404267e 23875714 PMC3807845

[15] Craig R. MCL1 provides a window on the role of the BCL2 family in cell proliferation, differentiation and tumorigenesis. Leukemia. 2002;16(4):444–454. 10.1038/sj.leu.2402416 11960321

[16] Rinkenberger JL, Horning S, Klocke B, Roth K, Korsmeyer SJ. Mcl-1 deficiency results in peri-implantation embryonic lethality. Genes Dev. 2000;14(1):23–27. 10640272 PMC316347

[17] Opferman JT, Letai A, Beard C, Sorcinelli MD, Ong CC, Korsmeyer SJ. Development and maintenance of B and T lymphocytes requires antiapoptotic MCL-1. Nature. 2003;426(6967):671–676. 10.1038/nature02067 14668867

[18] Hinds M, Smits C, Fredericks-Short R, Risk J, Bailey M, Huang D, et al Bim, Bad and Bmf: intrinsically unstructured BH3-only proteins that undergo a localized conformational change upon binding to prosurvival Bcl-2 targets. Cell Death Differ. 2007;14(1):128–136. 10.1038/sj.cdd.4401934 16645638

[19] Henriques J, Skepö M. Molecular dynamics simulations of intrinsically disordered proteins: on the accuracy of the TIP4P-D water model and the representativeness of protein disorder models. J Chem Theory Comput. 2016;12(7):3407–3415. 10.1021/acs.jctc.6b00429 27243806

[20] Best RB, Zheng W, Mittal J. Balanced protein–water interactions improve properties of disordered proteins and non-specific protein association. J Chem Theory Comput. 2014;10(11):5113–5124. 10.1021/ct500569b 25400522 PMC4230380

[21] Piana S, Donchev A, Robustelli P, Shaw D, et al Water Dispersion Interactions Strongly Influence Simulated Structural Properties of Disordered Protein States. J Phys Chem B. 2015;119(16):5113–5123. 10.1021/jp508971m 25764013

[22] Rauscher S, Gapsys V, Gajda MJ, Zweckstetter M, de Groot BL, Grubmüller H. Structural ensembles of intrinsically disordered proteins depend strongly on force field: a comparison to experiment. J Chem Theory Comput. 2015;11(11):5513–5524. 10.1021/acs.jctc.5b00736 26574339

[23] Turjanski AG, Gutkind JS, Best RB, Hummer G. Binding-induced folding of a natively unstructured transcription factor. PLoS Comput Biol. 2008;4(4):e1000060 10.1371/journal.pcbi.1000060 18404207 PMC2289845

[24] Lu Q, Lu HP, Wang J. Exploring the mechanism of flexible biomolecular recognition with single molecule dynamics. Phys Rev Lett. 2007;98(12):128105 10.1103/PhysRevLett.98.128105 17501161

[25] Law SM, Gagnon JK, Mapp AK, Brooks CL. Prepaying the entropic cost for allosteric regulation in KIX. Proc Natl Acad Sci U S A. 2014;111(33):12067–12072. 10.1073/pnas.1405831111 25002472 PMC4143015

[26] Ganguly D, Otieno S, Waddell B, Iconaru L, Kriwacki RW, Chen J. Electrostatically accelerated coupled binding and folding of intrinsically disordered proteins. J Mol Biol. 2012;422(5):674–684. 10.1016/j.jmb.2012.06.019 22721951 PMC3432731

[27] Ganguly D, Chen J. Topology-based modeling of intrinsically disordered proteins: Balancing intrinsic folding and intermolecular interactions. Proteins: Struct, Funct, Bioinf. 2011;79(4):1251–1266. 10.1002/prot.22960 21268115

[28] Baker CM, Best RB. Insights into the binding of intrinsically disordered proteins from molecular dynamics simulation. WIREs Comput Mol Sci. 2014;4(3):182–198. 10.1002/wcms.1167 PMC8336759 34354764

[29] Karanicolas J, Brooks CL. The origins of asymmetry in the folding transition states of protein L and protein G. Protein Sci. 2002;11(10):2351–2361. 10.1110/ps.0205402 12237457 PMC2373711

[30] Rogers JM, Oleinikovas V, Shammas SL, Wong CT, De Sancho D, Baker CM, et al Interplay between partner and ligand facilitates the folding and binding of an intrinsically disordered protein. Proc Natl Acad Sci U S A. 2014;111(43):15420–15425. 10.1073/pnas.1409122111 25313042 PMC4217413

[31] Koga N, Takada S. Folding-based molecular simulations reveal mechanisms of the rotary motor F1–ATPase. Proc Natl Acad Sci U S A. 2006;103(14):5367–5372. 10.1073/pnas.0509642103 16567655 PMC1459361

[32] Okazaki Ki, Koga N, Takada S, Onuchic JN, Wolynes PG. Multiple-basin energy landscapes for large-amplitude conformational motions of proteins: Structure-based molecular dynamics simulations. Proc Natl Acad Sci U S A. 2006;103(32):11844–11849. 10.1073/pnas.0604375103 16877541 PMC1567665

[33] Lu Q, Wang J. Single molecule conformational dynamics of adenylate kinase: energy landscape, structural correlations, and transition state ensembles. J Am Chem Soc. 2008;130(14):4772–4783. 10.1021/ja0780481 18338887

[34] Wang Y, Gan L, Wang E, Wang J. Exploring the dynamic functional landscape of adenylate kinase modulated by substrates. J Chem Theory Comput. 2012;9(1):84–95. 10.1021/ct300720s 26589012

[35] Noel JK, Whitford PC, Sanbonmatsu KY, Onuchic JN. SMOG@ ctbp: simplified deployment of structure-based models in GROMACS. Nucleic Acids Res. 2010;38(suppl 2):W657–W661. 10.1093/nar/gkq498 20525782 PMC2896113

[36] Clementi C, Nymeyer H, Onuchic JN. Topological and energetic factors: what determines the structural details of the transition state ensemble and “en-route” intermediates for protein folding? An investigation for small globular proteins. J Mol Biol. 2000;298(5):937–953. 10.1006/jmbi.2000.3693 10801360

[37] Levy Y, Cho SS, Onuchic JN, Wolynes PG. A survey of flexible protein binding mechanisms and their transition states using native topology based energy landscapes. J Mol Biol. 2005;346(4):1121–1145. 10.1016/j.jmb.2004.12.021 15701522

[38] Wang J, Wang Y, Chu X, Hagen SJ, Han W, Wang E. Multi-Scaled Explorations of Binding-Induced Folding of Intrinsically Disordered Protein Inhibitor IA3 to its Target Enzyme. PLoS Comput Biol. 2011;7(4):e1001118 10.1371/journal.pcbi.1001118 21490720 PMC3072359

[39] Biarnés X, Pietrucci F, Marinelli F, Laio A. METAGUI. A VMD interface for analyzing metadynamics and molecular dynamics simulations. Comput Phys Commun. 2012;183(1):203–211.

[40] Kumar S, Rosenberg JM, Bouzida D, Swendsen RH, Kollman PA. The weighted histogram analysis method for free-energy calculations on biomolecules. I. The method. J Comput Chem. 1992;13(8):1011–1021. 10.1002/jcc.540130812

[41] Huang Y, Liu Z. Kinetic advantage of intrinsically disordered proteins in coupled folding–binding process: a critical assessment of the “fly-casting” mechanism. J Mol Biol. 2009;393(5):1143–1159. 10.1016/j.jmb.2009.09.010 19747922

[42] Chebaro Y, Ballard AJ, Chakraborty D, Wales DJ. Intrinsically Disordered Energy Landscapes. Sci Rep. 2015;5:10386–10397. 10.1038/srep10386 25999294 PMC4441119

[43] Gasteiger E, Hoogland C, Gattiker A, Duvaud S, Wilkins MR, Appel RD, et al Protein identification and analysis tools on the ExPASy server. Springer; 2005 10.1385/1-59259-890-0:571 10027275

[44] Uversky VN, Gillespie JR, Fink AL. Why are “atively unfolded” proteins unstructured under physiologic conditions? Proteins: Struct, Funct, Bioinf. 2000;41(3):415–427. 10.1002/1097-0134(20001115)41:3<415::aid-prot130>3.0.co;2-7 11025552

[45] Das RK, Pappu RV. Conformations of intrinsically disordered proteins are influenced by linear sequence distributions of oppositely charged residues. Proc Natl Acad Sci U S A. 2013;110(33):13392–13397. 10.1073/pnas.1304749110 23901099 PMC3746876

[46] Zerze GH, Best RB, Mittal J. Sequence-and Temperature-Dependent Properties of Unfolded and Disordered Proteins from Atomistic Simulations. J Phys Chem B. 2015;119(46):14622–14630. 10.1021/acs.jpcb.5b08619 26498157 PMC6141988

[47] Müller-Späth S, Soranno A, Hirschfeld V, Hofmann H, Rüegger S, Reymond L, et al Charge interactions can dominate the dimensions of intrinsically disordered proteins. Proc Natl Acad Sci U S A. 2010;107(33):14609–14614. 10.1073/pnas.1001743107 20639465 PMC2930438

[48] Shoemaker BA, Portman JJ, Wolynes PG. Speeding molecular recognition by using the folding funnel: the fly-casting mechanism. Proc Natl Acad Sci U S A. 2000;97(16):8868–8873. 10.1073/pnas.160259697 10908673 PMC16787

[49] Levy Y, Onuchic JN, Wolynes PG. Fly-casting in protein-DNA binding: frustration between protein folding and electrostatics facilitates target recognition. J Am Chem Soc. 2007;129(4):738–739. 10.1021/ja065531n 17243791

[50] Ganguly D, Zhang W, Chen J. Electrostatically accelerated encounter and folding for facile recognition of intrinsically disordered proteins. PLoS Comput Biol. 2013;9(11):e1003363 10.1371/journal.pcbi.1003363 24278008 PMC3836701

[51] Schreiber G, Haran G, Zhou HX. Fundamental aspects of protein- protein association kinetics. Chem Rev. 2009;109(3):839–860. 10.1021/cr800373w 19196002 PMC2880639

[52] Blöchliger N, Xu M, Caflisch A. Peptide binding to a PDZ domain by electrostatic steering via nonnative salt bridges. Biophys J. 2015;108(9):2362–2370. 10.1016/j.bpj.2015.03.038 25954893 PMC4423040

[53] Day CL, Smits C, Fan FC, Lee EF, Fairlie WD, Hinds MG. Structure of the BH3 domains from the p53-inducible BH3-only proteins Noxa and Puma in complex with Mcl-1. J Mol Biol. 2008;380(5):958–971. 10.1016/j.jmb.2008.05.071 18589438

[54] Pettersen EF, Goddard TD, Huang CC, Couch GS, Greenblatt DM, Meng EC, et al UCSF Chimera: a visualization system for exploratory research and analysis. J Comput Chem. 2004;25(13):1605–1612. 10.1002/jcc.20084 15264254

[55] Noel JK, Whitford PC, Onuchic JN. The shadow map: a general contact definition for capturing the dynamics of biomolecular folding and function. J Phys Chem B. 2012;116(29):8692–8702. 10.1021/jp300852d 22536820 PMC3406251

[56] Lammert H, Schug A, Onuchic JN. Robustness and generalization of structure-based models for protein folding and function. Proteins: Struct, Funct, Bioinf. 2009;77(4):881–891. 10.1002/prot.22511 19626713

[57] Miyazawa S, Jernigan RL. Residue–residue potentials with a favorable contact pair term and an unfavorable high packing density term, for simulation and threading. J Mol Biol. 1996;256(3):623–644. 10.1006/jmbi.1996.0114 8604144

[58] Cho SS, Levy Y, Wolynes PG. Quantitative criteria for native energetic heterogeneity influences in the prediction of protein folding kinetics. Proc Natl Acad Sci U S A. 2009;106(2):434–439. 10.1073/pnas.0810218105 19075236 PMC2626720

[59] Azia A, Levy Y. Nonnative electrostatic interactions can modulate protein folding: molecular dynamics with a grain of salt. J Mol Biol. 2009;393(2):527–542. 10.1016/j.jmb.2009.08.010 19683007

[60] Givaty O, Levy Y. Protein sliding along DNA: dynamics and structural characterization. J Mol Biol. 2009;385(4):1087–1097. 10.1016/j.jmb.2008.11.016 19059266

[61] Chu X, Wang Y, Gan L, Bai Y, Han W, Wang E, et al Importance of electrostatic interactions in the association of intrinsically disordered histone chaperone Chz1 and histone H2A. Z-H2B. PLoS Comput Biol. 2012;8(7):e1002608 10.1371/journal.pcbi.1002608 22807669 PMC3395605

