# Supplementary figures and images for: Role of non-native electrostatic interactions in the coupled folding and binding of PUMA with Mcl-1
Source: PLoS Comput Biol. 2017 Apr 3;13(4):e1005468. doi: 10.1371/journal.pcbi.1005468 (PMC5400261; doi:10.1371/journal.pcbi.1005468)

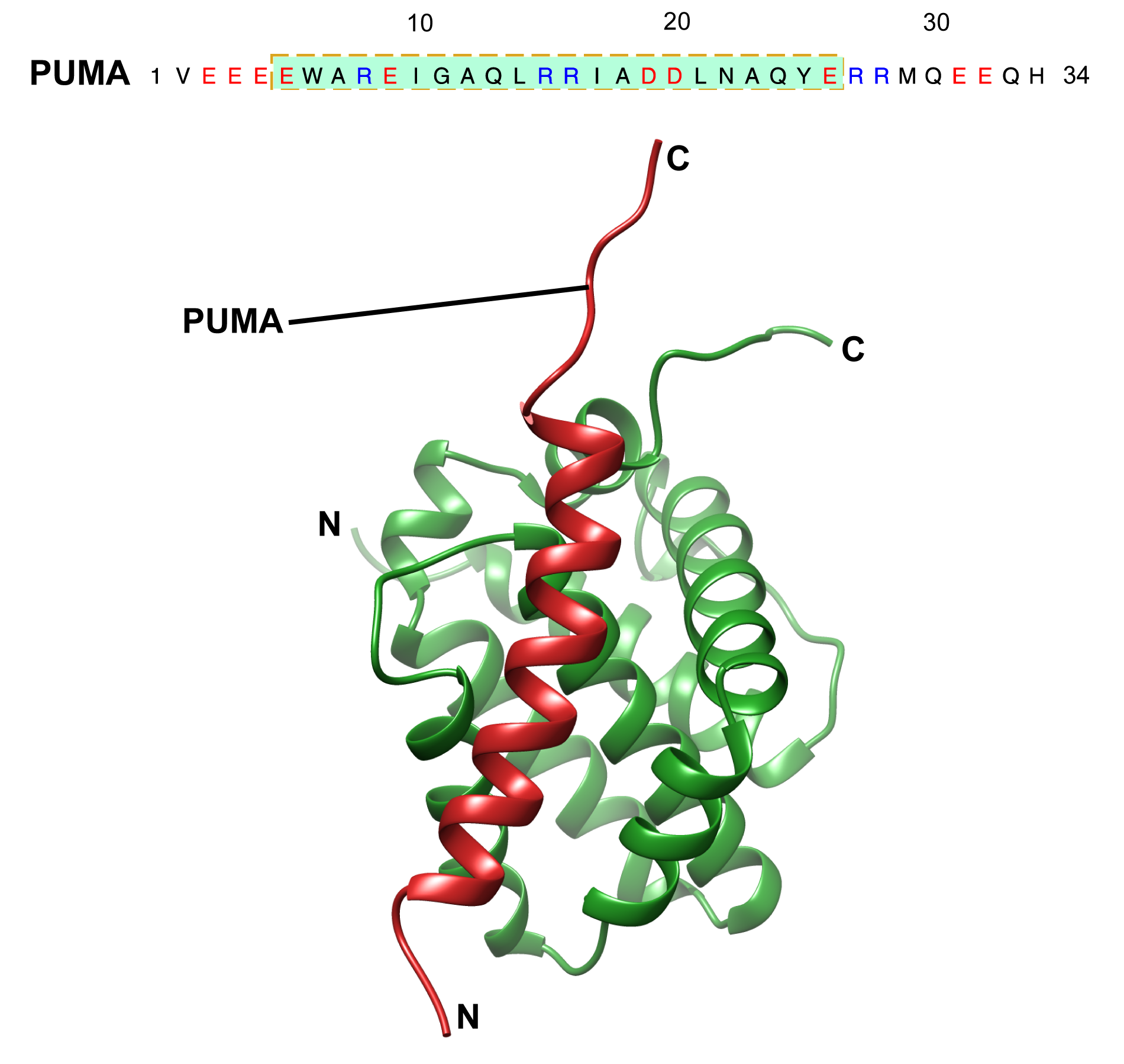

Supplement: S1 Fig — Upper: the sequence of full-length PUMA (34 a.a.) used in the simulations. Positive charged and negative charged residues are colored in blue and red. The region of helix in bound state is shown in light green box. Lower: The Mcl-1 · PUMA complex with full-length PUMA, constructed based on the NMR structure 2ROC (27 a.a. PUMA). Mcl-1 and PUMA are shown in green and red cartoons, respectively. (TIF) [file pcbi.1005468.s001.tif]

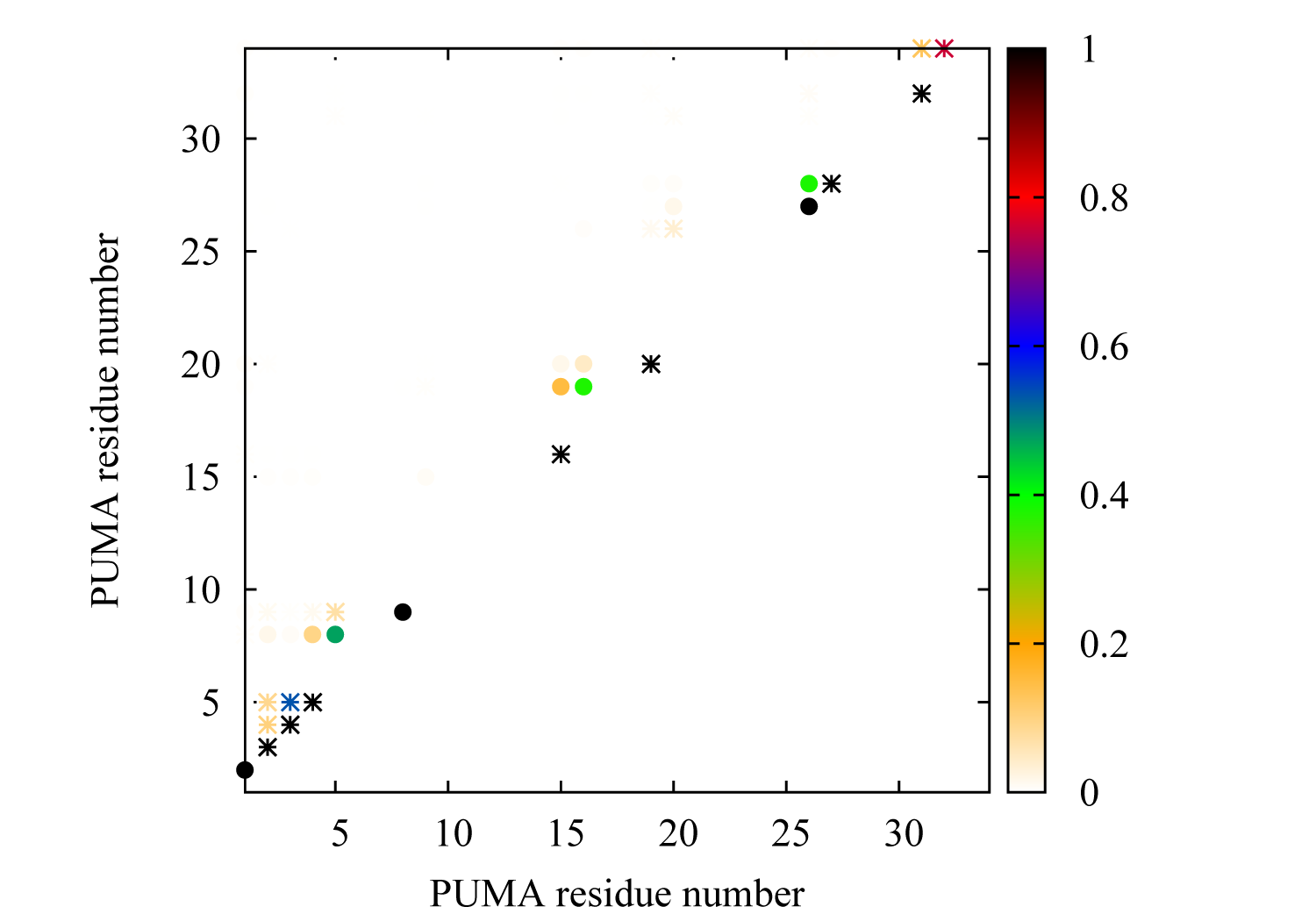

Supplement: S2 Fig — Attractive and repulsive contacts are depicted by dots and asterisks, respectively. (TIF) [file pcbi.1005468.s002.tif]

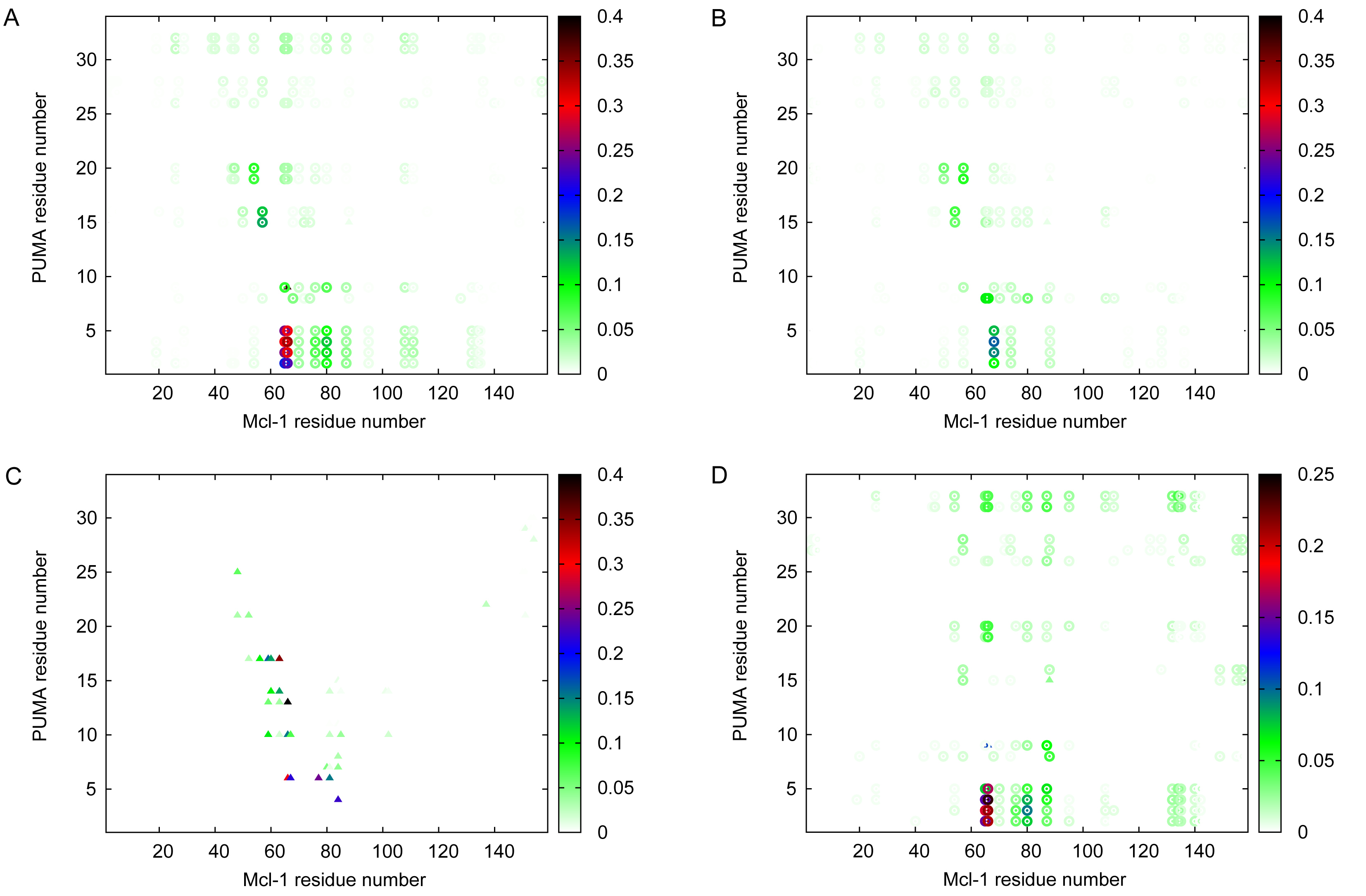

Supplement: S3 Fig — Contact map of opposite-charged (A), same-charged (B), and vdw (C) interactions within encounter complex at 10 mM ionic strength, as well as the contact map of opposite-charged interactions within the beginning of the evolution part (D). The beginning of evolution is collected for the complexes with only 1-2 number of inter contacts in the evolution part. The cutoff of the contact distance is 10.0 Å. Native contacts are illustrated as triangles, non-native contacts are illustrated as circles. (TIF) [file pcbi.1005468.s003.tif]

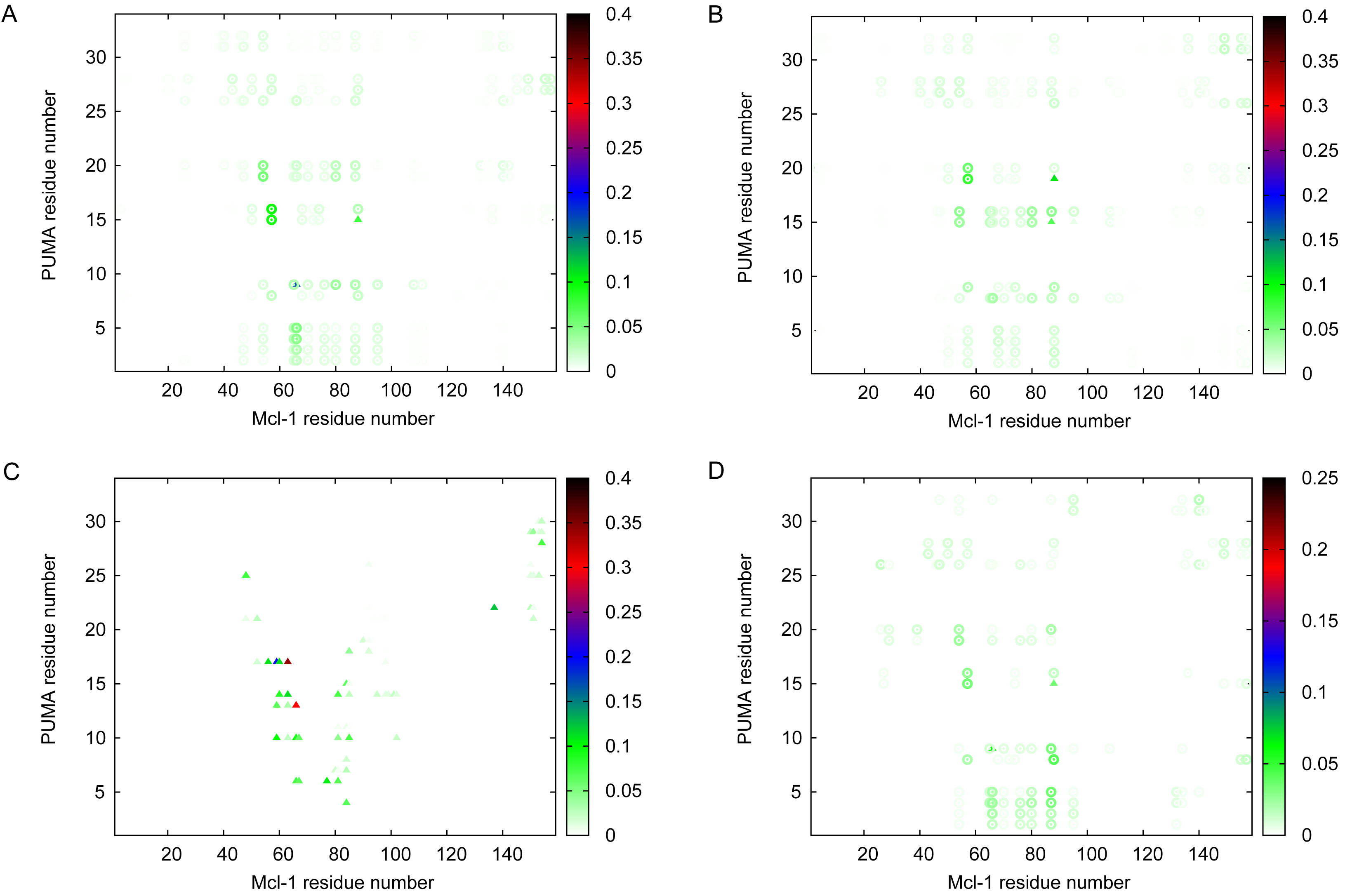

Supplement: S4 Fig — Contacts are more evenly distributed within the encounter complex for simulations performed without electrostatic forces included. Contact map of opposite-charged (A), same-charged (B), and vdw (C) interactions within encounter complex, as well as the contacp map of opposite-charged interactions within the beginning of the evolution part (D). All other parameters are the same as simulations performed with charges and IS = 10 mM. (TIF) [file pcbi.1005468.s004.tif]

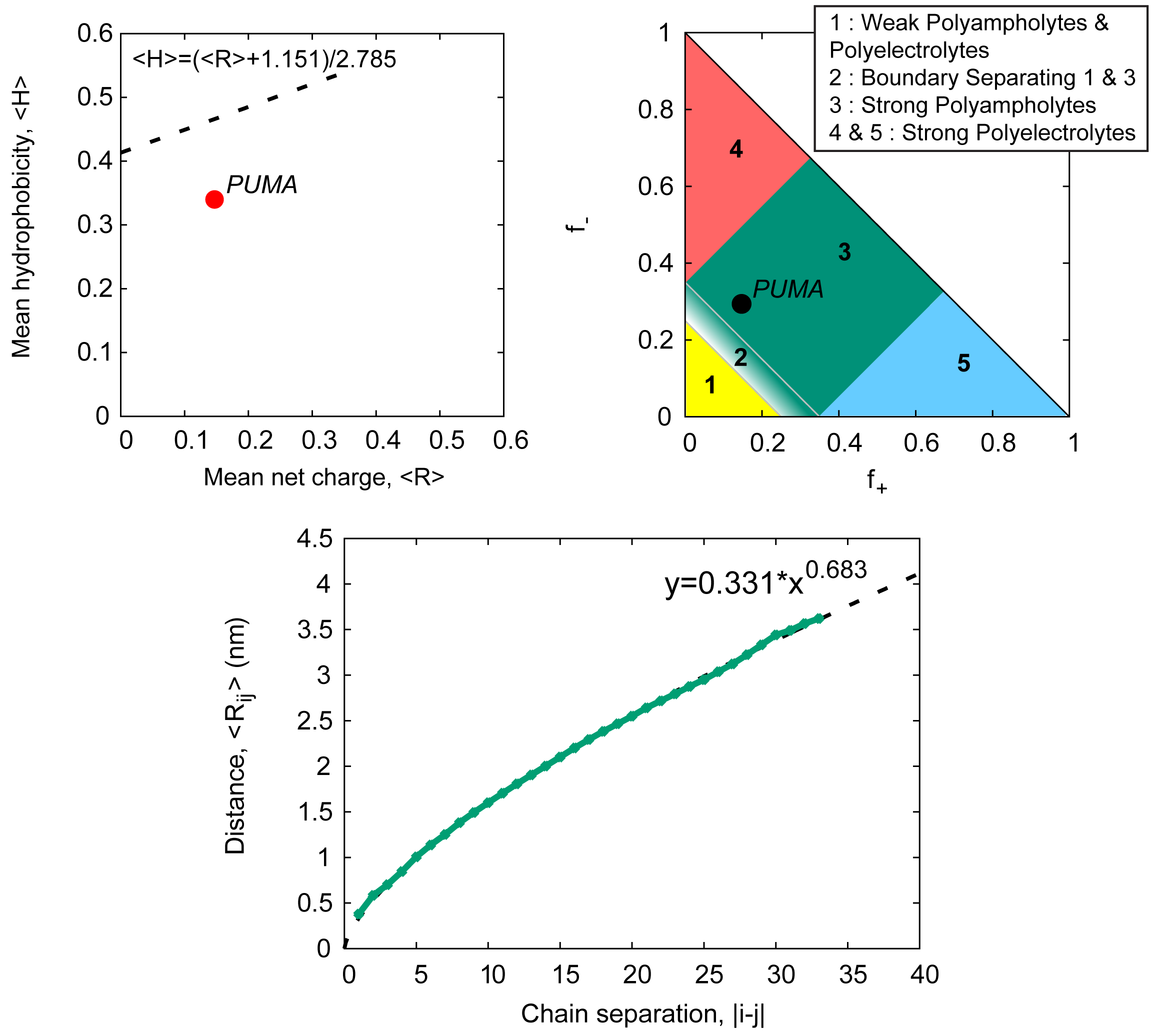

Supplement: S5 Fig — Top left: Uversky diagram [44] of IDPs (under dashed line) and globular proteins (above dashed line), Top right: Das and Pappu diagram [45] of IDPs. Bottom: Intra-chain distance (Rij) profiles of PUMA with respect to chain separation. Theoretical polymer scaling limit and fitting function are labeled. (TIF) [file pcbi.1005468.s005.tif]
