# Supplementary material for: Role of non-native electrostatic interactions in the coupled folding and binding of PUMA with Mcl-1
Source: PLoS Comput Biol. 2017 Apr 3;13(4):e1005468. doi: 10.1371/journal.pcbi.1005468 (PMC5400261; doi:10.1371/journal.pcbi.1005468)
Supplement: S1 File — (PDF) [file pcbi.1005468.s006.pdf]

Supplementary Materials:
Role of Non-Native Electrostatic Interactions in the Coupled Folding and Binding of PUMA with Mcl-1

Wen-Ting Chu[1], Jane Clarke[2], Sarah L. Shammas[2,3*] and Jin Wang[1,4*]

[1]*State Key Laboratory of Electroanalytical Chemistry, Changchun Institute of Applied Chemistry, Chinese Academy of Sciences, Changchun, Jilin, 130022, China*

[2]*Department of Chemistry, University of Cambridge, Lensfield Road, Cambridge, CB2 1EW, United Kingdom*

[3]*Department of Biochemistry, University of Oxford, South Parks Road, Oxford, OX1 3QU, United Kingdom*

[4]*Department of Chemistry & Physics, State University of New York at Stony Brook, Stony Brook, NY, 11794, USA*

[*]*Corresponding Author: sarah.shammas@bioch.ox.ac.uk; jin.wang.1@stonybrook.edu*

1 Methods

1.1 Calibrating the CG model

For the structure-based model run with reduced units, we calibrated the simulation temperature firstly. We ran REMD simulations with 48 replicas ranging from 29.12 K to 200.43 K to determine the folding temperature T_f. According to the specific heat curve calculated by REMD runs, the T_f of Mcl-1 is 154 K, which corresponds to the melting temperature (57 °C) of Mcl-1 in experiment[1]. Therefore, simulation temperature 129 K will be comparable to the room temperature (298 K).

The native nonbonded potential can be separated into intra- and inter-molecular terms:

$$V_{nonbonded}^{native} = \alpha V_{intra}^{native} + \beta V_{inter}^{native} \quad \text{(ESI-1)}$$

The energy of intramolecular interactions within PUMA were rescaled by altering the α parameter of PUMA to match the helical content in the experiments. 1 μs simulation were performed to calibrate α. The fraction of helix was calculated from the number of consecutive torsions. In our simulation, a residual helix requires at least 3 consecutive torsions between 30° and 120°, similar as the settings in previous simulations[1,2]. The alpha helical content of PUMA in complex (pdb 2ROC) is about 70%, which is consistent with the experimental data (about 65%)[1]. In experiments, the alpha helical content of PUMA in unbound state is about 20%, which corresponds to 0.7 in α.

Finally, the energy of intermolecular interaction between Mcl-1 and PUMA were rescaled by altering the β parameter between Mcl-1 and PUMA to match the dissociation constant (K_d) in experiment. The reported K_d of Mcl-1 and PUMA complex is 0.69 nM[3], which means that the binding energy between Mcl-1 and PUMA is about -7.39 kT. Metadynamics runs determined the β parameter to be 0.9.

1.2 Molecular Dynamics Simulations

All simulations were performed with Gromacs 4.5.5[4]. The coarse grained molecular dynamics simulations (CGMD) used Langevin equation with constant friction coefficient $\gamma = 1.0$. The cutoff for nonbonded interactions was set to 3.0 nm, and all bonds were constrained using LINCS algorithm[5].

The MD time step was set to 2.0 fs and the trajectories were saved every 2 ps. To enhance the sampling of binding events, a strong harmonic potential was added if the distance between the center of mass of the two chains of complex is greater than 6 nm. These conditions correspond to an effective protein concentration of 3.6 mM..

REMD simulations[6] were performed to determine the folding temperature of Mcl-1. 48 parallel replicas with temperature ranging from 29.12 K to 200.43 K ensure an efficient sampling. Each replica was performed for 1×10^9 MD steps. The exchanges were attempted every 5000 steps.

For thermodynamical simulations, well-tempered bias-exchange (WTBE) metadynamic runs were performed to overcome the high energy barriers between bound and unbound states of Mcl-1 and PUMA complex[7–10]. We define a native contact is formed if the Cα-Cα distance between any given native atom pair is within 1.2 times of its native distance. The native distance is calculated from the initial structure model. In WTBE run, 2 replicas, one with bias on the intermolecular contact and one with no bias (neutral) were performed in parallel. The fraction of intermolecular native contact number (Q_{inter}), fraction of intramolecular native contact number (Q_{intra}), and the helix content were collected as CV1, CV2, and CV3 in the metadynamic runs, respectively. A Gaussian of height 0.5 kJ/mol was added every 1 ps to the bias potential for all the walkers. The bias factor of well-tempered run was set to 10.0. Finally, WHAM[11] was applied to construct the energy landscape of binding-unbinding process.

For kinetics simulations, we ran 200 individual molecular simulations, each started from varying unbound configurations at 129 K simulation temperature, mimicking room temperature. During each simulation, the unbound state is the initial state, having no intermolecular contacts ($Q_{inter} = 0$). An encounter complex (EC) is defined once one or more native contacts are formed ($Q_{inter} > 0$); these are loosely bound states formed by capture events. The EC proceeds either to escape to the unbound state ($Q_{inter} = 0$), or form the bound state ($Q_{inter} \sim 0.7$). Simulations were ended upon reaching the bound state[12,13]. The mean passage time (MPT) of capture process (from unbound state to EC state, MPT_{cap}), first passage time (FPT) of evolution process (from EC state to bound state, FPT_{evo}), and first passage time (FPT) of binding-on process (from unbound state to bound state, FPT_{on}) were collected to compare the rate of binding in different conditions.

References

[1] Rogers JM, Oleinikovas V, Shammas SL, Wong CT, De Sancho D, Baker CM, et al. Interplay between partner and ligand facilitates the folding and binding of an intrinsically disordered protein. Proceedings of the National Academy of Sciences. 2014;111(43):15420–15425.

[2] De Sancho D, Best RB. Modulation of an IDP binding mechanism and rates by helix propensity and non-native interactions: Association of HIF1α with CBP. Molecular BioSystems. 2012;8(1):256–267.

[3] Rogers JM, Steward A, Clarke J. Folding and binding of an intrinsically disordered protein: fast, but not ”diffusion-limited”. Journal of the American Chemical Society. 2013;135(4):1415–1422.

[4] Hess B, Kutzner C, Van Der Spoel D, Lindahl E. GROMACS 4: algorithms for highly efficient, load-balanced, and scalable molecular simulation. Journal of chemical theory and computation. 2008;4(3):435–447.

[5] Hess B, Bekker H, Berendsen HJ, Fraaije JG, et al. LINCS: a linear constraint solver for molecular simulations. Journal of computational chemistry. 1997;18(12):1463–1472.

[6] Sugita Y, Okamoto Y. Replica-exchange molecular dynamics method for protein folding. Chemical physics letters. 1999;314(1):141–151.

[7] Baftizadeh F, Cossio P, Pietrucci F, Laio A. Protein folding and ligand-enzyme binding from bias-exchange metadynamics simulations. Current Physical Chemistry. 2012;2(1):79–91.

[8] Marinelli F, Pietrucci F, Laio A, Piana S. A kinetic model of trp-cage folding from multiple biased molecular dynamics simulations. PLoS Comput Biol. 2009;5(8):e1000452.

[9] Biarnés X, Pietrucci F, Marinelli F, Laio A. METAGUI. A VMD interface for analyzing metadynamics and molecular dynamics simulations. Computer Physics Communications. 2012;183(1):203–211.

[10] Barducci A, Bussi G, Parrinello M. Well-tempered metadynamics: A smoothly converging and tunable free-energy method. Physical review letters. 2008;100(2):020603.

[11] Grossfield A. WHAM: the weighted histogram analysis method. Disponıvel em:¡ http://membrane urmc rochester edu/content/wham. 2012;.

[12] Huang Y, Liu Z. Kinetic advantage of intrinsically disordered proteins in coupled folding–binding process: a critical assessment of the ”fly-casting” mechanism. Journal of molecular biology. 2009;393(5):1143–1159.

[13] Chu X, Liu F, Maxwell BA, Wang Y, Suo Z, Wang H, et al. Dynamic Conformational Change Regulates the Protein-DNA Recognition: An Investigation on Binding of a Y-Family Polymerase to Its Target DNA. PLoS Computational Biology. 2014;10(9):e1003804.
